# Postoperative complications and oncologic outcomes after multimodal therapy of localized high risk soft tissue sarcoma

**DOI:** 10.1186/s13014-022-02166-4

**Published:** 2022-12-21

**Authors:** Vlatko Potkrajcic, Jonas Kolbenschlag, Saskia Sachsenmaier, Adrien Daigeler, Ruth Ladurner, Alexander Golf, Cihan Gani, Daniel Zips, Frank Paulsen, Franziska Eckert

**Affiliations:** 1 Department of Radiation Oncology, Eberhard-Karls-University Tuebingen, Hoppe-Seyler-Str. 3, 72076 Tübingen, Germany; 2 German Cancer Research Center (DKFZ), German Cancer Consortium (DKTK) Partnersite Tuebingen, Heidelberg, Germany; 3 Department of Orthopaedic Surgery, Eberhard-Karls-University Tuebingen, Hoppe-Seyler-Str. 3, 72076 Tübingen, Germany; 4 Department of Hand, Plastic, Reconstructive and Burn Surgery, BG Unfallklinik, Eberhard-Karls-University Tuebingen, Schnarrenbergstraße 95, 72076 Tübingen, Germany; 5 Department of General, Visceral and Transplant Surgery, Eberhard-Karls-University Tuebingen, Hoppe Seyler-Str. 3, 72076 Tübingen, Germany; 6 Department of Internal Medicine, Medical Oncology and Pulmonology, Eberhard-Karls-University Tuebingen, Otfried-Müller-Straße 14, 72076 Tuebingen, Germany; 7 Department of Radiation Oncology, Comprehensive Cancer Center, Medical University Vienna, Waehringer Guertel 18-20, 1090 Vienna, Austria; 8 Department of Radiation Oncology and Radiotherapy, Charité University Hospital, Charitépl. 1, 10117 Berlin, Germany

**Keywords:** Radiotherapy, Soft tissue sarcoma, Multimodal therapy, Postoperative complications

## Abstract

**Background:**

Standard therapy for localized high-risk soft tissue sarcoma includes surgical resection and neoadjuvant or adjuvant radiation therapy (± chemotherapy and locoregional hyperthermia). No difference in oncologic outcomes for patients treated with neoadjuvant and adjuvant radiation therapy was reported, whereas side effect profiles differ. The aim of this analysis was to analyse oncologic outcomes and postoperative complications in patients treated with multimodal treatment.

**Methods:**

Oncologic outcomes and major wound complications (MWC, subclassified as wound healing disorder, infection, abscess, fistula, seroma and hematoma) were evaluated in 74 patients with localized high-risk soft tissue sarcoma of extremities and trunk undergoing multimodal treatment, and also separately for the subgroup of lower extremity tumors. Clinical factors and treatment modalities (especially neoadjuvant vs. adjuvant radiotherapy) were evaluated regarding their prognostic value and impact on postoperative wound complications.

**Results:**

Oncologic outcomes were dependent on number of high risk features (tumor size, depth to superficial fascia and grading), but not on therapy sequencing (however with higher risk patients in the neoadjuvant group). Different risk factors influenced different subclasses of wound healing complications. Slightly higher MWC-rates were observed in patients treated with neoadjuvant therapy, compared to adjuvant radiotherapy, although only with a trend to statistical significance (31.8% vs. 13.3%, *p* = 0.059). However, except for wound infections, no significant difference for other subclasses of postoperative complications was observed between neoadjuvant and adjuvant therapy. Diabetes was confirmed as a major risk factor for immune-related wound complications.

**Conclusion:**

Rates of major wound complications in this cohort are comparable to published data, higher rates of wound infections were observed after neoadjuvant radiotherapy. Tumor localization, patient age and diabetes seem to be major risk factors. The number of risk factors for high risk soft tissue sarcoma seem to influence DMFS. Neoadjuvant treatment increases the risk only for wound infection treated with oral or intravenous antibiotic therapy and appears to be a safe option at an experienced tertiary center in absence of other risk factors.

## Introduction

Soft tissue sarcomas (STS) are a rare and heterogenous group of malignancies derived from connective tissue [1], accounting for about 1% of all adult malignancies [2, 3]. For most localized high-risk extremity STS, previous radical concepts (limb amputation) have been gradually replaced with more moderate approaches as a part of multimodal therapy [4–6]. Thus, surgical resection represents a cornerstone and the essential treatment component, with an objective of complete resection while preserving the maximal function [7]. In high-risk situations, surgical treatment is complemented with other therapy modalities including radiotherapy as well as chemotherapy and hyperthermia in selected cases [7, 8]. After demonstrating equal survival rates for patients treated with limb-sparing surgical resection (combined with radiation therapy) compared to patients treated with amputation, the use of radiation therapy in multimodal treatment of STS has been established [9]. Furthermore, compared to limb-sparing surgery only, various studies demonstrated better local control (LC) rates for patients treated with additional radiation therapy [9, 10], which might translate into improved overall survival (OS) [10, 11].

Regarding the timing of radiation therapy, no difference in OS rates between patients with STS treated with neoadjuvant or adjuvant radiation therapy was demonstrated in most retrospective studies [12–14] as well as in a randomized controlled trial [15]. Contrary to these findings, a recent pooled analysis of published literature demonstrates a survival benefit in patients with extremity STS treated with adjuvant radiation therapy (compared to neoadjuvant therapy). However, these results might be influenced by selection and sampling bias as well as imbalanced risk factors between the groups [16].

A difference in acute and late side-effect profiles between patients treated with neoadjuvant or adjuvant radiation therapy has been observed in various studies. The rates of postoperative complications after neoadjuvant radiation therapy in patients with STS are relatively consistent throughout literature. Most studies did not evaluate risk factors for individual postoperative complication subgroups, but rather investigated postoperative complications in general. Even though therapy modalities varied among the studies, significantly higher rates of postoperative complications were observed in patients treated with neoadjuvant therapy [15–20]. However, these complications seem to be non-progressive with time [15] and are usually manageable with pharmacological or surgical intervention [17]. Furthermore, higher risk for late toxicity is connected with adjuvant therapy [14, 21], due to higher radiation dose and larger volumes compared to neoadjuvant therapy [14].

The timing of radiation therapy to achieve the best survival rates with less complications and good functional outcomes is controversial and it seems that there is no consensus about the optimal timing. The goal of this study was to evaluate oncologic outcomes and assess postoperative wound complications (as well as patient, tumor and treatment characteristics which could influence them) in patients with localized high-risk STS undergoing multimodal treatment.

## Materials and methods

In a single institution analysis, clinical records of patients with localized STS were retrospectively collected and analysed. The study protocol was approved by the local Ethics Committee (Nr 399/2020BO). Included were adult patients with histologically confirmed and localized STS who underwent curative multimodal treatment (at least surgery and radiation therapy) between 2011 and 2017. Patients with uterine, retroperitoneal and head and neck STS were excluded. According to tumor localization, patients were divided into three groups: lower extremities (including the groin), upper extremities (including the axilla) and trunk (including abdominal, pelvic, thoracic and breast area, retroperitoneum excluded). Staging was carried out with contrast enhanced MRI or CT of the tumor-region and at least CT of the lungs.

Multimodal therapy consisted of surgical resection and radiation therapy (either intensity-modulated radiation therapy-IMRT or 3D conformal radiation therapy-3DCRT). Sequential and concomitant chemotherapy was applied in younger patients with high-risk STS. Concomitant hyperthermia was applied in selected cases as an additional therapy modality. The standard radiation therapy dose was 45.0–50.4 Gy delivered in 25–28 fractions for neoadjuvant therapy and complemented by boost with 10–16 Gy in 5–8 fractions for adjuvant therapy. Radiation treatment planning was based on a planning CT using individual patient positioning. Target volumes were delineated by the aid of diagnostic imaging using Monaco planning system, Version 5.11.03 or Oncentra Masterplan treatment planning system 4.3 (both Elekta AB, Stockholm, Sweden), following recommendations for radiotherapy of high-risk soft tissue sarcomas [22]. Treatment planning (optimization) for IMRT was performed by the above-mentioned version of Monaco or the inhouse product Hyperion 2.4.5 and by above-mentioned version of Oncentra for 3D-RT. Planning and optimization for intensity modulated radiotherapy was based on an algorithm for fluence modulated, inverse treatment planning using biological objectives. Treatment was delivered by 6/15 MV Elekta linear accelerators with positioning controls using cone-beam CT or portal imaging. Concomitant therapy with ifosfamide was delivered in two cycles for both neoadjuvant and adjuvant therapy (3000 mg/m^2^ on day 1 and 2, as well as on day 21 and 22 of the irradiation). Sequential chemotherapy was applied in younger patients with high-grade tumors following the IAWS-protocol [23], using the combination of ifosfamide (3000 mg/m^2^ on day 1–3) and doxorubicin (60 mg/m^2^ on day 1) every 22 days for up to 3–6 cycles. Locoregional hyperthermia was delivered twice a week during radio(chemo-)therapy using the MR-controlled partial body hyperthermia, regional deep or superficial hyperthermia (BSD 2000/3 D MRI, Pyrexar Medical, formerly BSD medical corporation, Salt Lake City, UT).

Postoperative complications were analysed retrospectively using imaging and clinical data. The definition of major wound complication (MWC) was based on and adapted from O´Sullivan et al. [15] and included wound healing disorder that required a secondary surgical procedure (debridement, drainage or secondary wound closure), wound infection or abscess with admission of oral or intravenous antibiotic therapy, postoperative seroma or hematoma where an invasive procedure was needed (surgery) and postoperative fistula. Additionally, we recorded minor wound complications in lower extremities: wound healing disorder treated with conservative therapy, as well as seroma or hematoma treated with aspiration or drainage. In order to perform an analysis in a more homogenous cohort and to exclude confounding factors of different rates of wound healing complications for different localizations after surgery (independently of radiotherapy), additional analysis was performed separately for patients with STS localized in lower extremity. Patients were rated as diabetic (type 1 or type 2) when diabetes was mentioned in the clinical charts and comorbidities and the patients were on anti-diabetic drugs or dependent on insulin.

Statistical analysis was performed with IBM SPSS Version 26. Survival times were estimated with the Kaplan Meier method and compared using the log-rank test. Multivariate analyses were carried out using the cox regression model. Fisher's exact test was used to describe correlations between categorized variables. Means were compared by two-sided Student’s t-test if the assumptions for the test were met. *p* value of less than 0.05 was defined as statistically significant. *p* value 0.05 to 0.1 was defined as a trend to statistical significance.

## Results

### Patient characteristics

A total of 74 patients were included in our analysis. Patient and treatment characteristics are presented in Table 1. Lower extremity was the most common localization (n = 39, 52.7%), followed by trunk (n = 20, 27.0%) and upper extremity (n = 15, 20.3%). Most patients showed at least two features of high-risk STS (intermediate or high grading according to FNCLCC, tumor size > 5 cm, subfascial localization). No patients with less than one high-risk tumor feature were included in the study. Undifferentiated pleomorphic sarcoma was the most frequent histological subtype (Table 1).

**Table 1:** Patient and tumor characteristics (Total n = 74)

	Whole cohort (n = 74)	Lower extremity (n = 39)
**Age (Years)**		
Mean	59.6	61.7
Range	18–87	36–81
**Sex**		
Female	36 (48.6%)	18 (46.2%)
Male	38 (51.4%)	21 (53.8%)
**Tumor size**		
⩽ 5 cm (T1)	13 (17.6%)	4 (10.3%)
> 5 cm (T2)	61 (82.4%)	35 (89.7%)
Mean tumor size (cm)	9.9 (± 5.5)	10.9 (± 5.9)
**Tumor depth to superficial fascia**		
Superficial (Ta)	13 (17.6%)	5 (12.9%)
Deep (Tb)	61 (82.4%)	34 (87.1%)
**Tumor malignancy grade**		
Grade I	2 (2.7%)	0 (0%)
Grade II	29 (39.2%)	16 (41.0%)
Grade III	41 (55.4%)	22 (56.4%)
Undetermined	2 (2.7%)	1 (2.6%)
**Number of high-risk features**		
3	49 (66.2%)	29 (74.3%)
2	18 (24.3%)	8 (20.5%)
1	5 (6.8%)	1 (2.6%)
Missing data	2 (2.7%)	1 (2.6%)
**Surgical margin status**		
R0	56 (75.7%)	30 (76.9%)
R1	16 (21.6%)	8 (20. 5%)
R2	2 (2.7%)	1 (2.6%)
**Histology**		
Undifferentiated pleomorphic sarcoma	25 (33.8%)	
Myxofibrosarcoma	9 (12.2%)	
Liposarcoma	8 (10.8%)	
Leiomyosarcoma	6 (8.1%)	
Synovial sarcoma	5 (6.8%)	
Other	21 (28.4%)	
**Histology (lower extremity)**		
Undifferentiated pleomorphic sarcoma		12 (30.8%)
Liposarcoma		5 (12.9%)
Synovial sarcoma		4 (10.1%)
Extrasceletal myxoid chondrosarcoma		3 (7.7%)
Other		15 (38.5%)
**Therapy characteristics**	Whole cohort	Lower extremity
Neoadjuvant radiotherapy	44 (59.5%)	24 (61.5%)
Adjuvant radiotherapy	30 (40.5%)	15 (38.5%)
Concomitant ifosfamide	43 (58.1%)	26 (66.7%)
No concomitant ifosfamide	31 (41.9%)	13 (33.3%)
Concomitant hyperthermia	42 (56.8%)	23 (59.0%)
No concomitant hyperthermia	32 (43.2%)	16 (41.0%)
Sequential chemotherapy	28 (37.8%)	16 (41.0%)
No sequential chemotherapy	46 (62.2%)	23 (59.0%)
3DCRT	56 (75.7%)	32 (82.1%)
IMRT	18 (24.3%)	7 (17.9%)

Analysing the whole patient cohort, median neoadjuvant and adjuvant radiotherapy doses were 50.4 Gy (range 45.0–51.0 Gy) and 66.0 Gy (range 50.0–66.0 Gy). In the group of patients treated with adjuvant radiotherapy, one patient had to abort radiotherapy due to flap-necrosis. The majority of patients received concomitant chemotherapy (n = 43, 58%). However, 5 patients received only the first cycle due to poor tolerance. Sequential chemotherapy was applied in 28 patients (38%) with median number of 4 cycles (range 3–5). Local or deep hyperthermia was applied in 42 patients (57%) with median number of 10 treatments (range 3–15).

### Oncologic outcomes

**Fig. 1:**
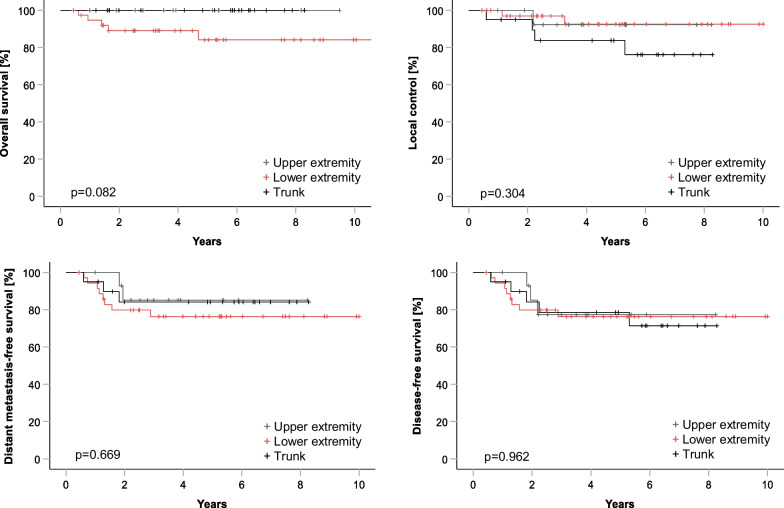
Kaplan–Meier curves demonstrating oncologic outcomes stratified by the tumor localization for the whole cohort. Worse OS-rates with trend to statistical significance were observed in patients with STS localized in lower extremity. No difference in LC, DMFS and DFS was observed between patient cohorts

Median follow-up for the whole cohort was 4.83 years (± 6.2 years). The 5-year rates for all patients were 91.9% (± 3.6%) for OS, 90.0% (± 3.9%) for LC, 80.4% (± 4.9%) for DMFS and 77.4% (± 5.2%) for DFS. Survival curves stratified by tumor localization are presented in Fig. 1. A trend to statistical significance for lower OS has been demonstrated for patients with STS localized in lower extremity (*p* = 0.082). The mean tumor size in patients with STS in lower extremity (according to pretherapeutic imaging) was larger compared to STS of other localizations: 10.9 ± 5.9 cm versus 8.5 ± 4.6 cm (*p* = 0.030). No other differences in distribution of risk factors (tumor depth to superficial, grading, number of high-risk features) between patients with STS stratified by tumor localization was detected (data not shown).

**Fig. 2:**
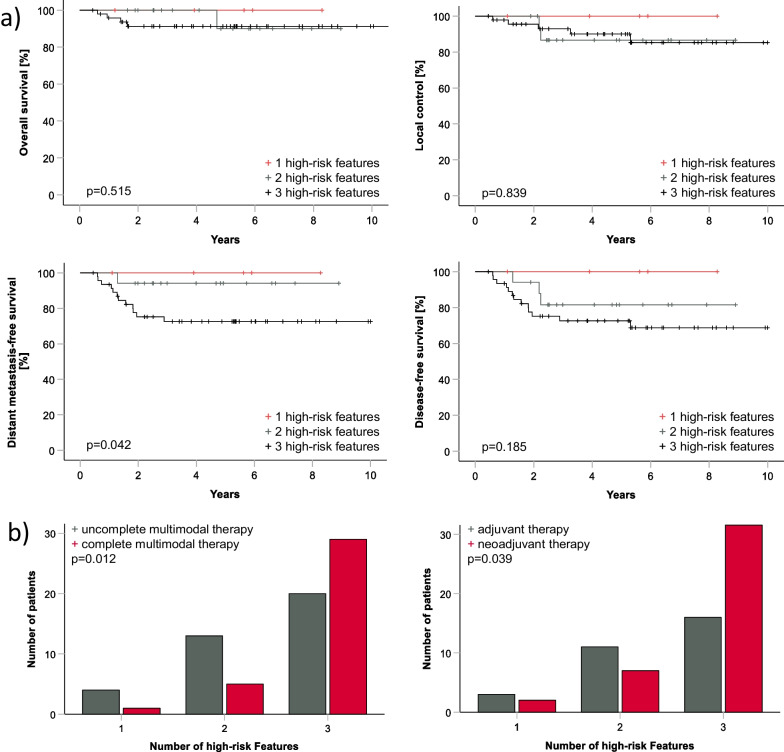
Panel a. Kaplan–Meier curves demonstrating oncologic outcomes stratified by number of tumor high-risk features for the whole cohort (patients with missing data excluded). Compared to patients with ≤ 2 high-risk features, worse DMFS-rates were observed in patients with all 3 high-risk STS features, no difference in OS, LC and DFS was shown. Panel b. Patients with all three high risk features were more often treated with neoadjuvant therapy, as well as with complete multimodal therapy (perioperative radiation therapy with both, concomitant chemotherapy and hyperthermia)

Stratified by the timing of radiation therapy (neoadjuvant vs. adjuvant), no difference in oncologic outcomes was observed (*p* = 0.328 for OS, *p* = 0.517 for LC, *p* = 0.386 for DMFS, *p* = 0.394 for DFS). However, unequal distribution of risk factors between these two groups was detected. Patients with all 3 high-risk features were more often treated with neoadjuvant therapy, compared to patients with only 1 or 2 high-risk features (*p* = 0.039). Furthermore, patients with all 3 high-risk features were more often treated with complete multimodal therapy, including both concomitant chemotherapy and hyperthermia in addition to perioperative radiation therapy (*p* = 0.012) (Fig. 2). Despite of the more intense treatment, worse DMFS-rates were observed in patients with all three tumor high-risk features (compared to patients with ≤ 2 high-risk features). No local recurrences, no metastases and no deaths were documented in patients with only 1 high-risk feature (Fig. 2).

### Wound complications and risk factors for the whole cohort

Overall MWC-rate for the whole cohort was 24.3% (n = 21). The most common complications were wound healing disorder and wound infection. Fisher’s exact test demonstrated unequal distribution of MWCs between patients with different anatomic localizations (*p* = 0.054, trend to statistical significance). Highest MWC-rates were observed in lower extremities (*p* = 0.013, compared to the rest of cohort). Upper extremity had the lowest MWC-rate (*p* = 0.066; trend to statistical significance). Additionally, individual wound complication subgroups were connected to specific tumor localization (Table 2).

**Table 2:** Major wound complications stratified by tumor localization

	Total	Lower extremity	Upper extremity	Trunk
Major wound complication	21/74 (24.3%)	**14/39 (35.9%)** **(*p* = 0.013)**	**1/15 (6.7%)** **(*p* = 0.066)**	3/20 (15.0%) (*p* = 0.364)
Wound healing disorder	11/74 (15.1%)	**10/39 (25.6%)** **(*p* = 0.007)**	**0/15 (0%)** **(*p* = 0.079)**	1/20 (5.0%) (*p* = 0.270)
Wound infection	11/74 (15.1%)	8/39 (20.5%) (*p* = 0.202)	1/15 (6.7%) (*p* = 0.326)	2/20 (10.0%) (*p* = 0.716)
Postoperative seroma	4/74 (5.5%)	**4/39 (10.2%)** **(*p* = 0.076)**	0/15 (0%) (*p* = 0.418)	0/20 (0%) (*p* = 0.570)
Fistula	4/74 (5.5%)	**4/39 (10.2%)** **(*p* = 0.076)**	0/15 (0%) (*p* = 0.418)	0/20 (0%) (*p* = 0.570)
Postoperative hematoma	2/74 (2.4%)	2/39 (5.1%) (*p* = 0.495)	0/15 (0%) (*p* = 0.651)	0/20 (0%) (*p* = 1.000)
Abscess	2/74 (2.4%)	2/39 (5.1%) (*p* = 0.495)	0/15 (0%) (*p* = 0.651)	0/20 (0%) (*p* = 1.000)

Total (n = 74). Each localization was compared to the rest of the cohort (bold text = singificant values or trend to significance)

In a first step, the relationship of relevant patient characteristics and clinical tumor variables with MWC was examined for the whole cohort. Older patients (> 65 years) had a trend to statistical significance for developing a MWC (*p* = 0.092). We found no correlation between tumor size > 5 cm and tumor depth to superficial fascia and MWC (*p* = 0.391 and *p* = 0.332, respectively). Furthermore, number of tumor high-risk features seems to have no influence on the risk for developing MWCs (*p* = 0.739). However, our findings demonstrate a strong correlation between diabetes and MWCs (*p* < 0.001), as well as with individual major complication subgroups: wound infection (*p* < 0.001), abscess (*p* = 0.008), fistula (*p* = 0.044) and wound healing disorder (*p* = 0.065; trend to statistical significance). No impact on postoperative hematoma or seroma was observed (*p* = 0.184 and *p* = 0.338, respectively). Furthermore, a correlation between postoperative seroma and fistula (*p* < 0.001) was observed. A trend to statistical significance for less wound healing disorders was demonstrated in upper extremities (*p* = 0.079). No other specific correlations for wound complications in patients with tumor localized in upper extremity and trunk were found, most probably due to the small number of events.

The impact of the timing of radiation therapy (neoadjuvant therapy vs. adjuvant therapy) on MWC was tested for the whole cohort. With trend to statistical significance, a higher MWC-rate was demonstrated in patients treated with neoadjuvant therapy (31.8% vs. 13.3%, *p* = 0.059). Furthermore, neoadjuvant therapy was associated with higher rate of wound infection (22.7% vs. 3.4%, *p* = 0.022). No correlation was found for wound healing disorder, abscess, seroma, hematoma and fistula (*p* = 0.103, *p* = 0.360, *p* = 0.478, *p* = 0.360 and *p* = 0.125, respectively). Patients with large tumors (> 5 cm) were more often treated with neoadjuvant therapy (*p* = 0.029).

No influence of concomitant chemotherapy (*p* = 1.000), concomitant hyperthermia (*p* = 0.787) or sequential chemotherapy (*p* = 0.783) on MWC was demonstrated. Furthermore, no statistically significant correlation was found between these therapy modalities and any subgroup of MWCs: wound healing disorder, wound infection, abscess, postoperative seroma, hematoma or fistula (data not shown). No influence of MWCs on oncologic outcomes was observed: OS (*p* = 0.912), LC (*p* = 0.679), DMFS (*p* = 0.625) and DFS (*p* = 0.384).

### Oncologic outcomes, wound complications and risk factors for lower extremities

Tumor localization in the lower extremity resulted in highest number of MWCs and also represents the largest anatomical subgroup in the whole cohort. For this reason, additional separate analyses of oncologic outcomes, wound complications and risk factors was performed for this patient group. The 5-year rates for patients with lower extremity STS were 84.1% (± 6.8%) for OS, 92.6% (± 5.2%) for LC, 76.3% (± 7.4%) for DMFS and 76.3% (± 7.4%) for DFS.

**Fig. 3:**
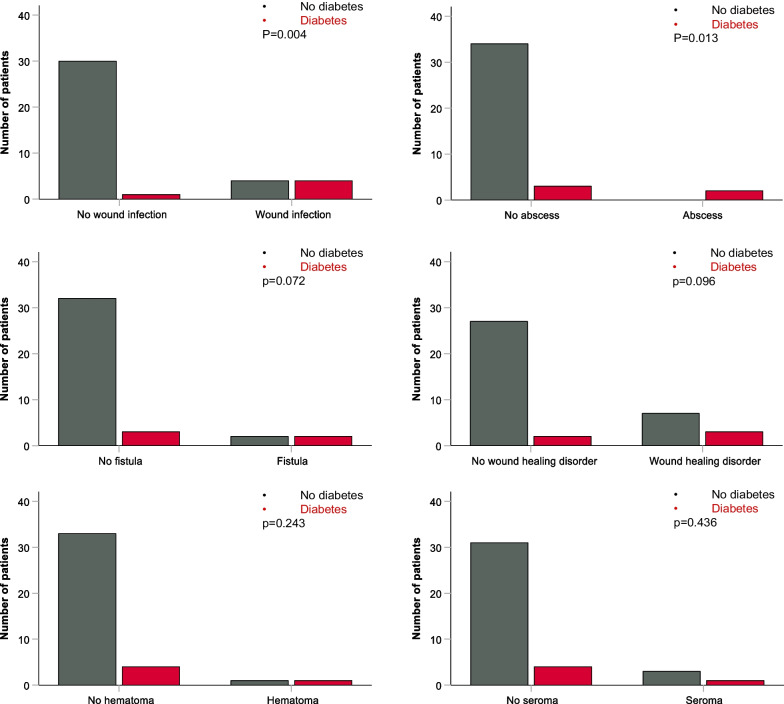
Bar graphs showing the influence of preexisting diabetes (diabetes mellitus type 1 or type 2) on major wound complications in patients with STS in lower extremity. Patients without diabetes had lower rate of wound infections and abscesses. A trend to statistical significance was observed for wound healing complication and fistula. No significant correlation between diabetes and seroma or hematoma was found

As already stated, localization in lower extremity was identified as a risk factor for developing MWCs, as well as for individual major complication subgroups: wound healing disorder, seroma and fistula (Table 2). Most of the tumors in this localization were > 5 cm (89.8%) and deep to superficial fascia (87.1%). Furthermore, patients with large tumors (> 5 cm) were with statistical significance more often treated with neoadjuvant therapy (*p* = 0.029). Concordant to results in the whole cohort, higher rate of MWCs (*p* = 0.098; trend to statistical significance) was observed in older patients (> 65 years) with STS localized in lower extremity. Deep localization was connected with MWCs with a trend to statistical significance (*p* = 0.092). In patients with tumor in lower extremity, diabetes was significantly associated with MWCs (*p* = 0.003), as well as with some individual complications (wound infection and abscess with statistical significance as well as with fistula and wound healing disorder with trend to statistical significance). All patients with diabetes developed at least one major postoperative complication (Fig. 3). Equal to results for the whole cohort, MWC seem to have no influence on survival rates in patients with STS in lower extremity: OS (*p* = 0.542), LC (*p* = 0.450), DMFS (*p* = 0.721), DFS (*p* = 0.721).

**Fig. 4:**
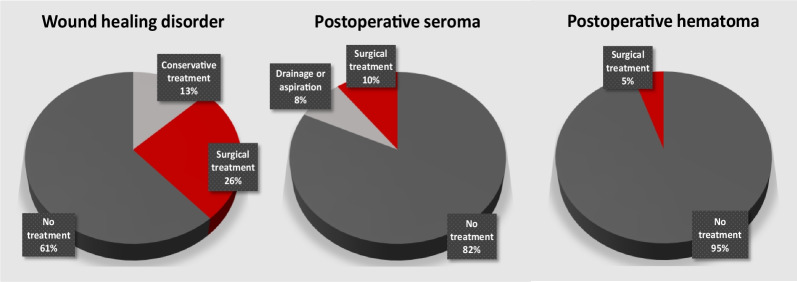
Pie charts demonstrating the distribution of minor and major wound complications for lower extremities, as well as the proportion of patients where no treatment regarding wound complications was needed (either no complication or patients with clinical or imaging finding where no treatment was needed). Most of the patients had either no complications or only imaging finding where no treatment was needed. Wound healing disorder was observed in 15 patients (38.5%). Conservative treatment was applied in 5/39 patients (12.8%), surgical treatment was needed in 10/39 patients (25.6%). In total, 7/39 patients had postoperative seroma (17.9%). A drainage or seroma aspiration was needed in 3/39 (7.7%) patients. Surgical intervention was needed in 4/39 (10.3%) patients (classified as major wound complication). Postoperative hematoma was documented in 2/39 patients (5.1%). Surgical treatment was performed in all patients with hematoma requiring intervention

The impact of the timing of radiation therapy on MWCs in patients with STS in lower extremity is demonstrated in Table 3. Slightly higher MWC rate in patients treated with neoadjuvant therapy were observed (*p* = 0.097, trend to statistical significance). Furthermore, wound infection was significantly more common after neoadjuvant therapy (Table 3). A correlation between postoperative seroma and fistula was observed in patients with STS in lower extremity (*p* = 0.002). Proportion of minor and major wound complications (for wound healing disorder, seroma and hematoma) was analyzed for lower extremities (Fig. 4).

**Table 3:** Major wound complications for lower extremities stratified by the timing of the treatment

	Neoadjuvant therapy (n = 24)	Adjuvant therapy (n = 15)	Significance
Patients with MWC	11 (45.8%)	3 (20.0%)	***p* = 0.097**
Total number of MWCs	27	3	
Wound healing disorder	8 (33.3%)	2 (13.3%)	*p* = 0.155
Wound infection	8 (33.3%)	0 (0.0%)	***p* = 0.012**
Abscess	2 (8.3%)	0 (0.0%)	*p* = 0.372
Postoperative seroma	3 (12.5%)	1 (6.7%)	*p* = 0.498
Postoperative hematoma	2 (8.3%)	0 (0.0%)	*p* = 0.372
Fistula	4 (16.7%)	0 (0.0%)	*p* = 0.129

Bold text significant values or trend to significance

## Discussion

To the best of our knowledge, this is the first analysis to identify patient, tumor and treatment related risk factors for different subclasses of MWCs in a general population of high-risk STS patients as well as for lower extremity STS. Not all subclasses of postoperative complications were influenced by timing of multimodal treatment. Only wound infection was significantly more common after neoadjuvant therapy. Number of high-risk features was identified as a prognostic marker for worse oncologic outcome regarding DMFS.

The MWC-rate in our study (24.3% in the whole cohort; 35.9% in lower extremities) was in line with the data from other studies that investigated postoperative complications after multimodal treatment for STS (both neoadjuvant and adjuvant radiotherapy, partly complemented by other modalities) [5, 15, 17–20]. Treatment modalities varied among studies and differences in definition of MWC have been detected. In most cases, the definition was adopted from the study published in 2002 by O´Sullivan et al. [15, 17, 19, 20, 24]. However, wound infections treated with intravenous antibiotic therapy without surgical intervention were excluded from the definition in one study [19]. Furthermore, differences in inclusion of postoperative seroma treated with drainage or aspiration have been detected. Higher MWC-rates in patients treated with neoadjuvant therapy (23.8–39.7%) compared to patients treated with adjuvant therapy (8.0–23.1%) were reported [5, 13, 15, 17–20]. Concordant to these data, our study demonstrated higher MWC-rates in patients treated with neoadjuvant therapy, compared to adjuvant therapy, although only with a trend to statistical significance (whole cohort: 31.8% vs. 13.3%, *p* = 0.059; lower extremities: 45.8% vs. 20.0%, *p* = 0.097). Furthermore, it seems that neoadjuvant therapy might be connected with some wound complication subgroups, as it shows correlation with wound infection (for both, the whole cohort and lower extremities). Due to low number of events for some wound complication subgroups, no statistical significance was reached. However, all hematoma, abscesses and fistula were observed in patients treated with neoadjuvant therapy.

Correlation between MWCs (and MWC subgroups) and specific patient, tumor and treatment characteristics was observed. Tumor localization in lower extremities was detected as a risk factor for MWCs in general (*p* = 0.013), as well as for individual complication subgroups (wound healing disorder, postoperative seroma and fistula). This observation is in line with published data, where lower extremity localization was identified as a risk factor for postoperative complications [15, 18, 25–27]. Thus, this localization was described as the strongest tumor-related predictor for wound complications, increasing the risk threefold compared to localization in upper extremity [28]. This observation can be supported by our findings, as the localization in upper extremity demonstrated lower risk for MWC (*p* = 0.066; trend to statistical significance). Furthermore, even though no statistical significance was reached, our data demonstrate that all postoperative seroma and hematoma as well as abscesses and fistula were located in lower extremity. These data suggest further evidence that tumor localization is a factor associated with postoperative wound complications and that additional risk factors for MWC shall be considered (e.g. functional load).

Older patients (> 65 years) had higher risk for MWCs. Regarding the impact of patient age, discrepant findings have been reported in the literature [28]. However, the clinical impact of age related changes in wound healing might be related to patients comorbidities rather than to age alone [29]. No impact of tumor size or depth and postoperative complications was found for the whole cohort in our study, possibly due to relatively small number of patients with small and superficial tumors in our cohort. However, in patients with STS in lower extremities, deep seated STS had higher risk for MWC (with a trend to statistical significance). Discrepant findings about tumor size and depth are published in the literature. Various cutoff points for tumor size have been used, which makes the results hard to compare [15, 18, 19, 30, 31]. Higher complication rates are reported in patients with large tumors (> 10 cm) [15, 32]. Furthermore, linear correlation between tumor diameter and MWC has been reported [19]. These findings suggest that by using different cutoff points, tumor size might increasingly affect wound complications. Further studies indicated a correlation between wound healing complications and resection volume [33], as well as with duration of the operation [34], which might indicate the correlation of postoperative complications with deep seated and large tumors.

Due to delayed response to injury and impaired function of immune cells [35], as well as prolonged inflammatory phase in the wound healing cascade [36], diabetes represents one of the most important causes of impaired wound healing. Furthermore, a strong correlation between diabetes and the risk of surgical site infection has been demonstrated [37]. In patients with STS, retrospective studies have identified preexisting diabetes as an independent risk factor for MWCs [19, 32]. These findings are supported by the results of our study as diabetes seems to be a strong predictive factor for postoperative complications in general. Furthermore, a correlation with wound complication subgroups connected with wound healing and immune response has been detected (wound infection and abscess with statistical significance, wound healing disorder and fistula with trend to statistical significance), whereabout no impact on postoperative seroma or hematoma was observed. All patients with diabetes in our study developed at least one MWC.

No difference in oncologic outcomes between patients with high-risk STS treated with neoadjuvant or adjuvant therapy was demonstrated in our analysis for the whole cohort, as well as in patients with STS in lower extremities. These findings are in line with published data [12–15], even though some reports might have been biased by selection of patients and show imbalance of risk factors. Patients with all three high-risk tumor features (size > 5 cm, deep localization, grading 2–3) were more often treated with neoadjuvant therapy in our study (*p* = 0.039), which might have put this group of patients to a higher risk for poor oncologic outcomes and might have underestimated the importance of the timing of radiation therapy. Thus, our results demonstrate worse oncologic outcomes regarding DMFS for patients with all three STS high-risk features. Furthermore, no local recurrences, distant metastases or deaths were observed in patients with only one high-risk feature. A trend to statistical significance for worse OS was observed in patients with STS in lower extremity. However, the mean tumor size in lower extremities was significantly larger, compared to the rest of the cohort. Thus, this finding might have put these patients to a higher risk for worse survival outcomes. The impact of tumor size, tumor depth and grading have been investigated in various studies. Tumor size > 5 cm and deep localization have been detected as risk factors for DMFS [38, 39], DFS [38] and OS [39]. However, these risk factors seem to have no impact on LC [38–42]. Furthermore, worse oncologic outcomes regarding DMFS [38, 41], OS [41, 42] and DFS [38] are connected with high-grade STS. The observation that patients with combination of more high-risk features are at higher risk for distant metastases was already noted in retrospective studies [39, 43]. Considering these findings, our data suggest that neoadjuvant therapy should be considered in the interdisciplinary treatment planning of high-risk STS and that the timing of radiation therapy might affect patient survival rates, as patients with unfavorable prognostic factors do not have inferior oncologic outcomes.

Our data suggest specific correlation between not only MWC in general, but also with individual subgroups of postoperative complications. These findings might be important in prevention and treatment planning of specific postoperative complications (e.g. adjustment of antibiotic regimens for diabetic patients at high risk of infection or consideration of flap reconstruction in large, deep-seated lower extremity tumors at high risk of seroma and fistula). A trend to statistical significance for higher MWC-rates, as well as statistically significant higher rates of wound infections were observed in patients treated with neoadjuvant therapy. However, published data suggest that these complications seem to be non-progressive with time [15] and are usually manageable with pharmacological or surgical intervention [17]. In line with published data [15], no impact of MWCs on oncologic outcomes was observed in our study. Furthermore, differences in acute and late toxicity between patients treated with different therapy modalities have been noted in the literature. Thus, the use of neoadjuvant therapy seems to be a safe strategy at large tertiary centers, especially in the absence of other risk factors such as diabetes. With the published lower rates of long-term morbidities after neoadjuvant treatment [14, 21], due to higher radiation dose and larger volumes needed in patients treated with adjuvant therapy [44, 45], neoadjuvant therapy should be considered during treatment planning of STS patients, especially in cases where high adjuvant radiation dose is hardly applicable.

## Data Availability

The datasets used and analysed during the current study are available from the corresponding author on reasonable request.

## Abbreviations

STS: Soft tissue sarcoma
LC: Local control
OS: Overall survival
CT: Computed tomography
MR(I): Magnetic resonance (imaging)
IMRT: Intensity modulated radiotherapy
3DCRT: 3-Dimensional conformal radiotherapy
IGRT: Image-guided radiation therapy
Gy: Gray
MWC: Major wound complication
DFS: Disease-free survival
DMFS: Distant metastasis-free survival
FNCLCC: Fédération Nationale des Centres de Lutte Contre le Cancer
